# Supplementary material for: Lower probability and shorter duration of infections after COVID-19 vaccine correlate with anti-SARS-CoV-2 circulating IgGs
Source: PLoS One. 2022 Jan 31;17(1):e0263014. doi: 10.1371/journal.pone.0263014 (PMC8803178; doi:10.1371/journal.pone.0263014)
Supplement: S1 File — (DOCX) [file pone.0263014.s001.docx]

**SUPPLEMENTARY MATERIAL**

**Supplementary Figures**

**S1 Figure. Workflow of our study pre- and post-vaccination**

**S2 Figure. Study design**


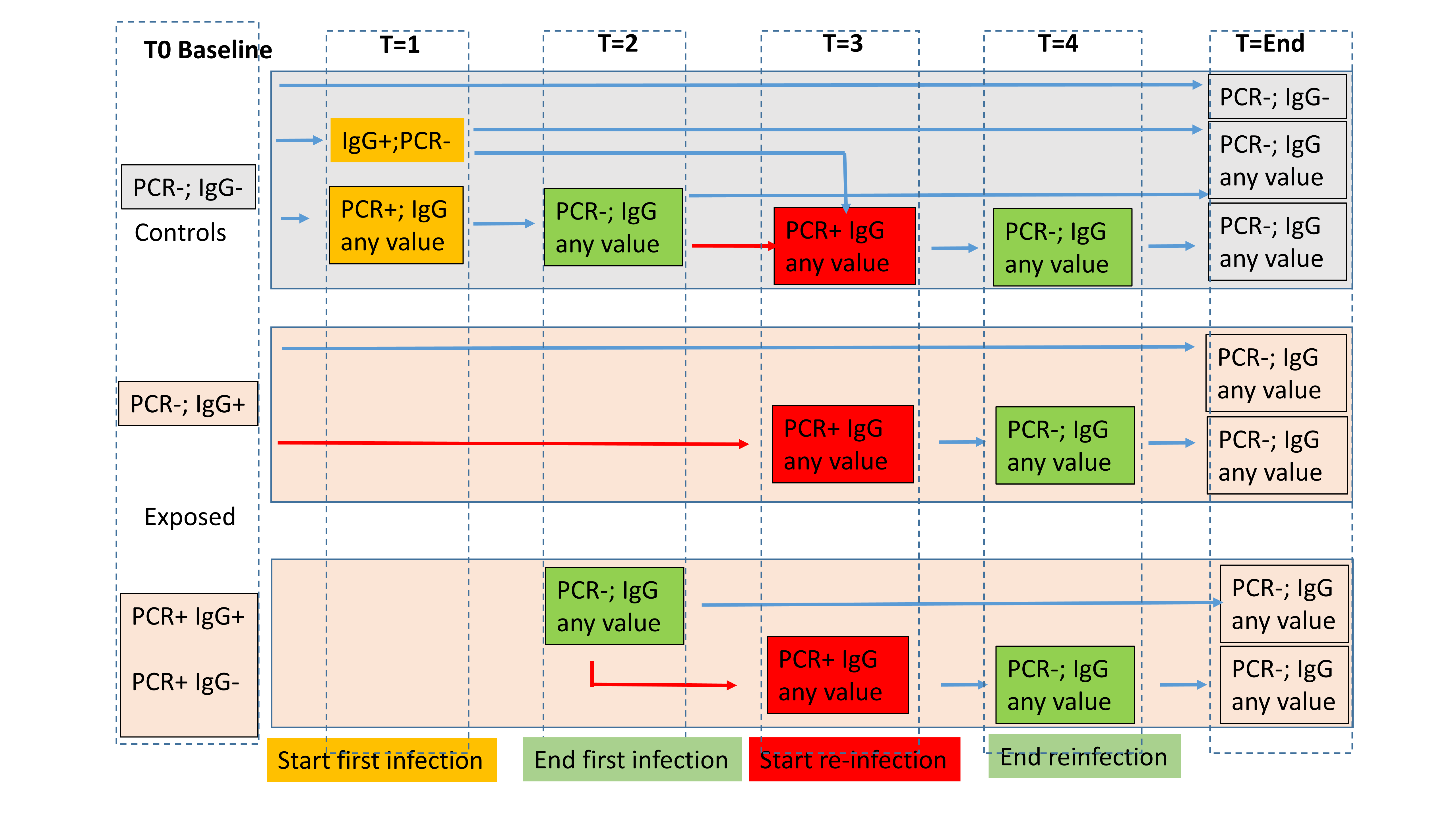


**Supplementary Tables**

**S1 Table. Descriptive characteristics of subjects included in pre- and post-vaccination cohorts**

|  |  | **Nr** | **%** | **Nr** | **%** |
| --- | --- | --- | --- | --- | --- |
|  |  | **(Tot=1493)** |  | **(Tot= 2029)** |  |
| **Age, Median (Q1-Q3)** |  | 41 (31-49) |  | 42 (31-51) |  |
| **Gender** | Female | 994 | 66.58 | 1324 | 65.25 |
|  | Male | 499 | 34.42 | 705 | 34.75 |
| **Profession** |  |  |  |  |  |
|  | Nurses/clinicians | 784 | 52.51% | 875 | 43.12% |
|  | Other | 709 | 47.49% | 1154 | 56.88% |

**S2 Table. Features of reinfections (positive swab)**

|  |  |  |  |  |  | **Viral genes at T0** | | | **Viral genes at first infection** | | |
| --- | --- | --- | --- | --- | --- | --- | --- | --- | --- | --- | --- |
| **ID** | **PCR status** | **IgG value** | **Duration first infection** | **Time to second infection** | **Duration second infection** | **E** | **RdRP** | **N** | **E** | **RdRP** | **N** |
| 1 | Negative | 0.45 | 77 | 43 | 7 |  |  |  | 36.78 | n | 37.57 |
| 2 | Negative | 0.68 | 52 | 188 | 4 |  |  |  | n | n | 37.97 |
| 3 | Negative | 0.86 | 41 | 80 | 18 |  |  |  | n | n | 36.49 |
| 4 | Negative | 0.91 | 70 | 220 | 2 |  |  |  | n | n | 37.65 |
| 5 | Negative | 1.18 | 73 | 161 | 2 |  |  |  | 32.3 | 36.68 | 31.25 |
| 6 | Positive | 0.39 | 7 | 34 | 43 | n | n | 38.04 | n | 37.67 | n |
| 7 | Positive | 0.05 | 154 | 50 | 7 | n | 35.99 | 34.68 | n | 37.46 | n |
| 8 | Positive | 0.50 | 108 | 126 | 4 | 31.14 | 32.62 | 31.14 | n | n | 38.8 |

n, not detectable

**S3 Table. Duration of infections and time from first and second infection (negativity)**

|  | **Median** | **Lower Quartile** | **Upper Quartile** | **P-value¥** |
| --- | --- | --- | --- | --- |
| **First infection** | 16.5 | 11 | 40.5 | <.0001 |
| **Reinfection** | 11 | 4 | 21 | 0.0035 |
| **Duration of negativity** | 34 | 21 | 85 |  |
| **Infection after vaccine** | 2 | 2 | 4 | Reference |

**¥** Wilcoxon rank tests

**S4 Table. Odd Ratio and 95% Confidence Intervals for the association with infection**

|  |  | **OR** | **Low 95%CI** | **Up 95%CI** | **P-values** | |
| --- | --- | --- | --- | --- | --- | --- |
| **Age** |  | 1.01 | 1.001 | 1.03 | 0.038 | |
| **Symptoms pre study** | **Yes vs No** | 1.37 | 0.96 | 1.96 | 0.087 | |
| **Profession** | **Nurse/Phisician vs other** | 1.90 | 1.44 | 2.51 | <.0001 | |
| **Swab at baseline** | **Neg vs Pos** | 0.46 | 0.11 | 1.89 | 0.279 |  |
| **IgG at baseline** | **>28 vs <28** | 0.34 | 0.15 | 0.80 | 0.014 |  |

OR from multivariable logistic models.
Legend OR=Odd ratio; considering IgG as continuous variable P=0.06.

**S5 Table. Descriptive characteristic of subjects included in the study pre-vaccination (n=1493)**

|  | **Categories** | **Nr** | **%** |
| --- | --- | --- | --- |
| **Personal History of Covid-19 Symptoms** | No | 1263 | 84.59 |
|  | Yes | 229 | 15.34 |
|  | Missing | 1 | 0.07 |
| **Flu between February and April 2020** | No | 1057 | 70.8 |
|  | Yes | 201 | 13.46 |
|  | Missing | 241 | 16.14 |
| **Contact with Covid-19 positive subjects in family** | No | 1170 | 78.37 |
|  | Yes | 54 | 3.62 |
|  | Missing | 267 | 17.01 |
| **Contact with Covid-19 positive at work** | No | 677 | 45.34 |
|  | Yes | 549 | 36.77 |
|  | Missing | 267 | 17.88 |
| **Contact with Covid-19 positive extra-work** | No | 1178 | 78.9 |
|  | Yes | 52 | 3.48 |
|  | Missing | 269 | 18.02 |
